# Lumasiran: a potential therapy for the management of primary hyperoxaluria type 1? An editorial

**DOI:** 10.1097/JS9.0000000000000143

**Published:** 2023-01-12

**Authors:** Sundus A. Ghani, Sheeba Burney, Fiza Muzaffar, Laiba Naseem, Hassan ul Hussain, Syeda T. Rehan, Hassan Mumtaz

**Affiliations:** a Dow University of Health Sciences, Karachi; b Health services Academy, Islamabad, Pakistan

## Background

Primary hyperoxaluria type 1 (PH1) is a rare autosomal recessive disorder, results from an inborn error of glyoxylate metabolism that is caused by deficiency of liver specific enzyme alanine glyoxylate aminotransferase (AGT). Mutations in the *AGXT* gene lead to dysfunction of vitamin B6-dependent AGT. This eventually leads to overproduction of oxalate with subsequent increased excretion from the kidney. Long-term complications of PH type 1 include progressive renal involvement and decline in glomerular filtration rate leading to urolithiasis and nephrocalcinosis^1^.

PH1 is estimated to be as prevalent as one to three cases per one million population and ~1 case per 120 000 live births in the Europe. Moreover, population of areas having consanguineous marriages are at a higher risk of developing PH, with 10% higher prevalence in North African and Middle Eastern nations^2^. Undifferentiated hyperoxaluria is seen in up to 43% of Pakistani pediatric stone patients, most likely due to consanguinity^3^.

## Conservative management

Managing PH1 currently involves conservative techniques like intensive hydration requiring gastrostomy or feeding tube in children and alkalization of urine through alkali citrate, potassium citrate, or pyrophosphate ions. Restriction of oxalate intake has been of limited effect^4^. Vitamin B6 is considered first-line therapy in PH1 patients that have B6-responsive mutations. Administration of pyridoxine has been shown to be associated with decrease in urine oxalate in 30% of patients with PH1^3^. But for decades, the only long-term option to correct the defective metabolism of glyoxylate in PH1 has been liver transplantation, sometimes in combination with kidney transplantation^5^.

## Novel regimen for PH1 and mechanism of action

After a recent clinical trial by Frishberg *et al* 6. The United States Food and Drug Administration (FDA) approved Lumasiran for treating PH1. It is administered subcutaneously and works on the principle of RNA interference (RNAi). Lumasiran is a synthetic small interfering RNA (siRNA) drug it inhibits the synthesis of glycolate oxidase, by cleaving the messenger RNA (mRNA) transcript which was encoded by the hydroxy acid oxidase 1 gene. Suppression of glycolate oxidase will lead to the accumulation of glycolate and decreased production of glyoxylate, an immediate precursor of oxalate, thus protecting from the injury caused by high oxalate levels^7^.

## Dosage and route of administration

In a randomized, placebo-controlled, phase ½ trial, consisting of 32 healthy participants and 20 PH1 patients. Healthy individuals received single doses of Lumasiran in ascending dose groups (0.3–6 mg/kg) in a ratio of 3 : 1 (Lumasiran: Placebo) and up to three doses of Lumasiran (1 or 3 mg/kg) were administered to PH1 patients at monthly or quarterly intervals^6^. The loading dose of Lumasiran is three doses of 6 and 3 mg/kg once monthly for patients weighing less than and more than 20 kg, respectively^8^.

## Pharmacokinetics

Pharmacokinetic profiles showed rapid systemic elimination indicating rapid hepatic uptake of the drug and urinary excretion was found to be a minor route of excretion of Lumasiran^6^.

## Efficacy and side effects

Different RCTs conducted showed that there was a decrease in 24-hour urinary oxalate excretion and urinary oxalate to creatinine ratio (UXo : Cr) while an increase in plasma and urinary glycolate levels. It also indicated improvement in nephrocalcinosis in some patients. Along with these beneficial effects, many side effects were also observed. Many participants experienced injection site pain, reactions, headache, nasopharyngitis, abdominal pain, nephrolithiasis, and rhinitis^6^,^9^–^11^. A detailed comparison of the efficacy and side effects of Lumasiran is provided in Table 1.

**Table 1:** Clinical Trials on Lumasiran.

References	Phase of trial	ClinicalTrial.gov identifier	Efficacy	Adverse effects
Frishberg *et al* 6.	1/2	NCT02706886	24-hour urinary oxalate excretion decreased up to 75%	Abdominal pain (18%) Headache (18%) Rhinitis (12%) Nephrolithiasis (12%) Cough (12%)
Hulton *et al* 9.	3	NCT03681184	Reduction in 24-hour urinary oxalate excretion (66.9%) after 3 mo and (64.1%) after 12 mo Improved nephrocalcinosis (13%)	ISR
Sas *et al* 10.	3	NCT03905694	Reduction in spot UXo : Cr in 6 mo (70.2%)	ISR (11.1%) Headache (5.5%)
Garrelfs *et al* 11.	3	NCT03681184	24-hour urinary oxalate excretion decreased up to 65.4%	ISR (38%) Erythema Pain Pruritus Discomfort

ISR indicates injection site reactions; UXo Cr, urinary oxalate to creatinine ratio.

## Future prospect and recommendations

Since PH1 is a chronic genetic disorder with increasing morbidity and mortality, treatment of the disease with the novel drug can reduce high-risk compilations like nephrolithiasis, dialysis, renal failure, kidney transplant, liver transplant, and a low standard of living. Additionally, the results from the clinical trials have proved Lumasiran to be a promising therapy in terms of efficacy and safety compared to other managements.

However, there is a dire need for further multiple large-scale trials to evaluate its long-term safety and efficacy. The drug is administered subcutaneously and since many people are not handy with an injection therefore a patient-friendly form of drug, which can be ingested orally, should be taken into consideration. Lastly, the cost of the drug should be lowered to make it affordable for people. The higher authorities should look into drug production and availability in underprivileged and developing countries to raise the standard of living.

## Ethical approval

Not applicable.

## Sources of funding

None.

## Authors’ contribution

S.A.G., S.B.: concept. F.M., L.N., S.T.R.: manuscript writing. H.M., H.u.H.: editing and review.

## Conflicts of interest disclosure

All authors have equally contributed to the manuscript and have approved the final manuscript to be published.

## Research registration unique identifying number (UIN)

None.

## Guarantor

Hassan Mumtaz.

## Data statement

Data will be made available upon request.
